# Robust Transgene Expression from Bicistronic mRNA in the Green Alga *Chlamydomonas reinhardtii*

**DOI:** 10.1534/g3.116.033035

**Published:** 2016-10-20

**Authors:** Masayuki Onishi, John R. Pringle

**Affiliations:** Department of Genetics, Stanford University School of Medicine, California 94305

**Keywords:** algae, bicistronic mRNA, IRES, transgene expression, translation reinitiation

## Abstract

The unicellular green alga *Chlamydomonas reinhardtii* is a model organism that provides an opportunity to understand the evolution and functional biology of the lineage that includes the land plants, as well as aspects of the fundamental core biology conserved throughout the eukaryotic phylogeny. Although many tools are available to facilitate genetic, molecular biological, biochemical, and cell biological studies in *Chlamydomonas*, expression of unselected transgenes of interest (GOIs) has been challenging. In most methods used previously, the GOI and a selectable marker are expressed from two separate mRNAs, so that their concomitant expression is not guaranteed. In this study, we developed constructs that allow expression of an upstream GOI and downstream selectable marker from a single bicistronic mRNA. Although this approach in other systems has typically required a translation-enhancing element such as an internal ribosome entry site for the downstream marker, we found that a short stretch of unstructured junction sequence was sufficient to obtain adequate expression of the downstream gene, presumably through post-termination reinitiation. With this system, we obtained robust expression of both endogenous and heterologous GOIs, including fluorescent proteins and tagged fusion proteins, in the vast majority of transformants, thus eliminating the need for tedious secondary screening for GOI-expressing transformants. This improved efficiency should greatly facilitate a variety of genetic and cell-biological studies in *Chlamydomonas* and also enable new applications such as expression-based screens and large-scale production of foreign proteins.

The unicellular green alga *Chlamydomonas reinhardtii* is a well established model organism that has been widely used for studies of photosynthesis, the structure and function of flagella and basal bodies, the cell cycle, and other processes (Harris 2001; Marshall 2008; Ostrowski *et al.* 2011; Heinnickel and Grossman 2013; Cross and Umen 2015). Its advantages include its haploid vegetative cells, a sequenced genome (Merchant *et al.* 2007), rapid growth under both auxotrophic and heterotrophic conditions, and well developed methods and resources for genetics, biochemistry, and microscopy. Genetic transformation into the *Chlamydomonas* nucleus has been used in many studies, and methods and reagents including promoters, terminators, enhancers, reporter genes, and auxotrophic and drug-resistance markers are available (for review, see Jinkerson and Jonikas 2015).

Nonetheless, expression of unselected transgenes, especially of heterologous origin, has remained a challenge in *Chlamydomonas*. In the most commonly used “two-promoter” approach, an unselected gene of interest (GOI) and a selectable marker are transcribed under the control of two separate promoter-terminator pairs (Figure 1A); the two expression modules are introduced either as separate DNA fragments or as a single cassette (Heitzer and Zschoernig 2007) and integrate into the genome at a random site(s) by nonhomologous end-joining. However, in many cases, ≤10% of the selected transformants coexpress the unselected GOI, presumably due to a low cotransformation rate of pairs of fragments, cleavage of two-gene cassettes with integration only of the fragment with the selectable marker (Zhang *et al.* 2014), and/or strong transcriptional and/or post-transcriptional silencing (Cerutti *et al.* 1997). Thus, a secondary screen—often rather tedious—for transformants expressing the GOI has typically been necessary. For example, we needed to screen >100 transformants to acquire a single clone that expressed the F-actin probe Lifeact-Venus at a level sufficient for visualization by fluorescence microscopy (Avasthi *et al.* 2014). These problems have handicapped cell biological studies and impeded development of expression-based screens, medium- to high-throughput imaging analyses, and the use of *Chlamydomonas* as a host for expression of foreign proteins.

**Figure 1:**
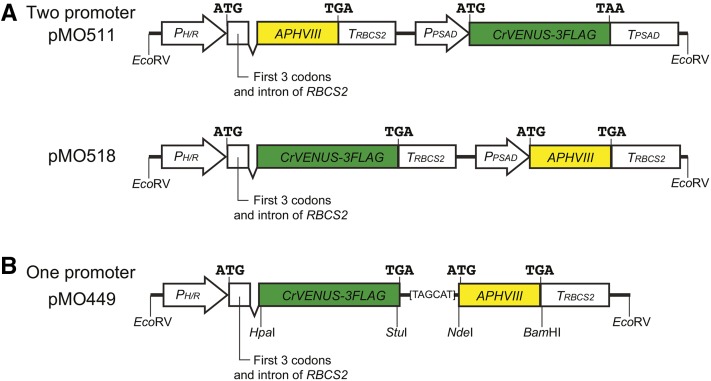
Representative two-promoter and one-promoter expression constructs as used in this study. All constructs were embedded in the identical plasmid backbone (outside of the *Eco*RV sites shown), as described in *Materials and Methods*. (A) Two-promoter constructs. Translation start and stop sites for each gene are shown. *P_H/R_*, strong hybrid *HSP70A/RBCS2* promoter; *APHVIII*, paromomycin-resistance gene; *T_RBCS2_*, *RBCS2* transcription terminator; *P_PSAD_*, strong *PSAD* promoter; *CrVENUS-3FLAG*, coding sequence of Venus codon-optimized for *Chlamydomonas* and tagged with three copies of the FLAG epitope; *T_PSAD_*, *PSAD* transcription terminator; *Eco*RV, restriction sites that can be used to excise the construct from the vector before transformation. Other restriction sites are present [*cf*. (B)] but not shown. (B) General structure of the one-promoter constructs used in this study as illustrated by that of plasmid pMO449. The 6-bp linker between the two ORFs is shown by the sequence of the transcribed strand; other symbols as in (A). The restriction sites shown can be used to replace the upstream GOI, the downstream selectable marker, or the linker sequence (see text).

In an alternative “one-promoter” approach, the constructs contain a single promoter and terminator that produce a bicistronic mRNA typically containing an upstream GOI and a downstream selectable marker with separate translation start and stop sites (Figure 1B). Expression of the selectable marker then requires transcription of the entire mRNA and thus expression of the GOI. This approach has been used previously for expression of introduced genes in some animals, cultured animal cells, fungi, and plants (Martínez-Salas 1999; Hunt and Maiti 2001). Translation of the GOI is usually mediated by canonical cap-dependent initiation (Hinnebusch and Lorsch 2012), whereas that of the selectable marker is controlled by cap-independent initiation mechanisms. Most vectors have employed an internal ribosome entry site (IRES) that is inserted in the untranslated region between the ORFs to enhance translation of the selectable marker. IRES elements are sequences of different lengths and secondary and tertiary structures that provide sites for the ribosome and translation-initiation factors to assemble independent of a 5′-cap structure. Originally identified in picornaviral genomes (Jang *et al.* 1988; Pelletier and Sonenberg 1988), IRES-mediated translation has now been found in various other viruses, as well as in some nonviral cellular mRNAs (Hellen and Sarnow 2001; Jackson 2013). Although, to our knowledge, there is no virus known to infect *Chlamydomonas*, and no IRES elements have been identified in its cellular mRNAs, some viral-derived IRES elements have been shown to function in organisms evolutionarily distant from the normal host species (Iizuka *et al.* 1994; Urwin *et al.* 2000; Dorokhov *et al.* 2002).

In a few cases, the IRES has been omitted, and the expression vectors have simply had the two ORFs of a bicistronic mRNA connected by a short stretch of unstructured sequence; such constructs have been used successfully in both plants and mammalian cells (Levine *et al.* 1991; Lough *et al.* 1997; Hunt and Maiti 2001). Although the precise mechanism of translation initiation for the downstream ORF in these constructs is not certain, it is thought to be through post-termination reinitiation (Skabkin *et al.* 2013) rather than leaky scanning or ribosome shunting (Jackson *et al.* 2012). Because of the inefficiency of post-termination reinitiation (Hunt and Maiti 2001; Jackson *et al.* 2012), expression of the downstream ORF by this mechanism would normally be expected to be less efficient than that obtained with an IRES.

In this paper, we describe expression vectors for *Chlamydomonas* that use a single promoter and transcription terminator to express a bicistronic mRNA encoding an upstream GOI and a downstream selectable marker without an intervening IRES; they achieve very efficient expression of the GOI in the majority of transformants.

## Materials and Methods

### Strains, media, and growth conditions

*Chlamydomonas reinhardtii* wild-type strain CC-124 (also known as 137c; Chlamydomonas Resource Center) was used in most experiments. Strain CMJ030 (also known as CC-4533; Zhang *et al.* 2014) was also used where indicated. Cells were grown in Tris-acetate-phosphate (TAP) medium (Gorman and Levine 1965) at 21–24° under constant illumination at 75 μmol photons m^−2^ sec^−1^. In most experiments, selection of transformants used paromomycin (Sigma) at a concentration from 10 to 40 µg/ml, as indicated. Some experiments used zeocin (InvivoGen) at 5 µg/ml, spectinomycin (Sigma) at 30 µg/ml, or hygromycin B (Sigma) at 5 µg/ml.

### Plasmid constructions

The plasmids used in this study are listed in Table 1 and Table 2. In construct descriptions, *P_XXX_* or *T_XXX_* indicates a promoter or terminator sequence from gene *XXX*; a hyphen indicates a fusion of two coding sequences or polypeptides (*e.g.*, *VENUS-3FLAG*); and a colon indicates a conjoinment of two DNA sequences that does not result in such a fusion (*e.g.*, *P_H/R_:APHVIII*). Plasmid constructions were performed using the one-step isothermal-assembly method (Gibson *et al.* 2009). Primer sequences are provided in Supplemental Material, File S1.

**Table 1:** Plasmids used in this study

Plasmid*^a^*	Promoter for GOI	GOI	Junction	Selectable Marker
pMO511*^b^*	*P_PSAD_*	*CrVENUS-3FLAG*	*P_H/R_*	*APHVIII*
pMO424*^b^*	*P_PSAD_*	*LifeAct-CrVENUS-3FLAG*	*P_H/R_*	*APHVIII*
pMO448	*P_H/R_*	N/A	N/A	*APHVIII*
pMO449	*P_H/R_*	*CrVENUS-3FLAG*	TAGCAT	*APHVIII*
pMO459	*P_H/R_*	*LifeAct-CrVENUS-3FLAG*	TAGCAT	*APHVIII*
pMO470	*P_H/R_*	*CrVENUS-3FLAG*	TGATAGCCAT	*APHVIII*
pMO471	*P_H/R_*	*CrVENUS-3FLAG*	TAGCCAT	*APHVIII*
pMO480	*P_H/R_*	*CrVENUS-3FLAG-CVIM**^c^*	TAGCAT	*APHVIII*
pMO482	*P_H/R_*	PRO1*-CrVENUS-FLAG*	TAGCAT	*APHVIII*
pMO483	*P_H/R_*	*IDA5-CrVENUS-3FLAG*	TAGCAT	*APHVIII*
pMO484	*P_H/R_*	*VFL2-CrVENUS-3FLAG*	TAGCAT	*APHVIII*
pMO488	*P_H/R_*	*CrVENUS*	TAGCAT	*APHVIII*
pMO490	*P_H/R_*	*sfGFP-3FLAG*	TAGCAT	*APHVIII*
pMO507	*P_PSAD_*	*CrVENUS-3FLAG*	TAGCAT	*APHVIII*
pMO508	*P_TUB2_*	*CrVENUS-3FLAG*	TAGCAT	*APHVIII*
pMO515	*P_H/R_*	*PMH1-CrVENUS-3FLAG*	TAGCAT	*APHVIII*
pMO518	*P_H/R_*	*CrVENUS-3FLAG*	*T_RBCS2_-P_PSAD_*	*APHVIII*
pMO519	*P_H/R_*	*CrmCHERRY*	TAGCAT	*APHVIII*
pMO520	*P_H/R_*	*CrmCHERRY-3FLAG*	TAGCAT	*APHVIII*

a Except for pMO511 (note *^b^* and Figure 1A), pMO424 (note *^b^*), and pMO518 (Figure 1A), all plasmids listed have the general structure shown for pMO449 in Figure 1B.

b In pMO511 (Figure 1A) and pMO424 (Avasthi *et al.* 2014), the selectable marker is upstream under the control of the *P_H/R_* promoter, and the GOI is downstream under the control of the *P_PSAD_* promoter. pMO424 is identical to pMO511 except that the *Chlamydomonas*-codon-optimized *Lifeact*-encoding sequence is fused at the N-terminus of *CrVENUS-3FLAG*.

c CVIM is expected to function as a “CAAX-box” for prenylation.

**Table 2:** Assessment of various sequences used as the junction between the GOI and the selectable marker

Row*^a^*	Plasmid*^b^*	Type of Junction*^c^*	Description or Sequence of Junction	No. of Transformants/µg DNA*^d^*	No. of Venus-Positive Clones*^e^*
1	pMO448	N/A	Control (no 5′ ORF as GOI)	∼2500	N/A
2	pMO518	Promoter-containing	Control (conventional two-promoter construct; see Figure 1A)	∼400*^f^*	∼1/16*^f^*
3	pMO455	IRES	EMCV (porcine encephalomyocarditis virus) IRES (586 bp)	39	0/16
4	pMO467	IRES	crTMV (crucifer-infecting tobamovirus) IRES-CP (148 bp)	13	1/16
5	pMO463	IRES	PVY (potato virus Y) long (91 bp)	35	0/16
6	pMO464	IRES	PVY short (66 bp)	21	0/16
7	pMO466	IRES	PFBV (Pelargonium flower break virus) (78 bp)	21	1/16
8	pMO473	IRES	PLRV (potato leafroll virus)*^g^*	64	10/16
9	pMO474	IRES	PLRV^mut11^*^h^*	73	8/16
10	pMO449	Non-IRES	TAGCAT*^i^*	327	15/16
11	pMO471	Non-IRES	TAGCCAT*^i^*	359	14/16
12	pMO470	Non-IRES	TGATAGCCAT*^i^*	452	13/16

a Using *CrVENUS-3FLAG* as a sample GOI and *APHVIII* (conferring resistance to paromomycin) as the selectable marker.

b For additional details, see Table 1 and/or *Materials and Methods*.

c With or without an IRES.

d Except for pMO518 (note *^f^*), the numbers of total and Venus-positive transformants shown here were taken from a single experiment that included all constructs; it used strain CMJ030 and the Bio-Rad electroporator with cells in in TAP medium plus 40 mM sucrose, followed by selection on TAP agar containing 20 µg/ml paromomycin. The numbers shown are consistent with those from multiple other experiments using strain CMJ030 or CC-124 with various subsets of the constructs.

e In each case, 16 transformants were examined by fluorescence microscopy for expression of the Venus protein.

f pMO518 was not included in the particular experiment that yielded the other data shown in this table. Thus, the numbers indicated are estimates based on several other experiments in which transformant numbers and numbers of GOI-positive transformants could be compared directly among pMO518, pMO448, and/or pMO449.

g *TAA* C **ATG** ATT **ATG** ACT CCG **ATG** AGG ATT ACG GTC TGG AGA GAG AGG CTG CAA CAA **ATG** *ATG*. Additional potential start codons, in frame with that of *APHVIII* itself, are in bold face; the *CrVENUS-3FLAG* stop codon and the *APHVIII* start codon are indicated by italics.

h *TAA* C **ATG** ATT **ATG** ACT CCG **ATG** AGG ATT ACG GTC TCC TCT CTC TCC CTG CAA CAA **ATG** *ATG*. Additional start codons, in frame with that of *APHVIII* itself, are in bold face; the *CrVENUS-3FLAG* stop codon and the *APHVIII* start codon are indicated by italics. The sequence altered by *mut11* is underlined (Jaag *et al.* 2003).

i Note (1) that the linkers contain one (pMO449 and pMO471) or two (pMO470) additional stop codons in frame with that of *Cr-VENUS-3FLAG* itself, and (2) that the *APHVIII* start codon is in frame with the stop codons in pMO449 but out of frame in pMO471 and pMO470.

Plasmid pMO511 (Figure 1A) was constructed by removing a *Hpa*I-flanked fragment from pLM004-Venus (Yang *et al.* 2014). Plasmid pMO448 was constructed by assembling two PCR products that were amplified from pLM004-Venus. First, primers MOP651/MOP653 were used to amplify the pUC19 backbone plus the *HSP70A/RBCS2* hybrid promoter (henceforth *P_H/R_*), the first three codons and first intron of *RBCS2*, and 29 additional base pairs of pLM004-Venus-derived sequence that include both *Hpa*I and *Stu*I sites. Second, primers MOP652/MOP654 were used to amplify the *APHVIII* coding sequence and the *RBCS2* 3′-UTR and *T_RBCS2_*; the fragment also includes 27 bp upstream of the *APHVIII* start codon that overlap with the sequence in the other fragment and provide consecutive TGA and TAG stop codons plus 3 bp of an *Nde*I site that is completed by the *APHVIII* start codon. To construct pMO449 (Figure 1B), a PCR fragment containing the codon-optimized *CrVENUS-3FLAG* was amplified from pLM004-Venus using primers MOP657/MOP658 and inserted into the *Hpa*I site of pMO448, maintaining reading frame with both the *RBCS2* start codon and the stop codons present in pMO448. To construct pMO470 and pMO471, preannealed oligonucleotides MOP696/MOP699 and MOP696/MOP698, respectively, were inserted between the *Stu*I and *Nde*I sites of pMO449. To construct pMO518 (Figure 1A), a PCR fragment containing a stop codon, *T_RBCS2_*, and *P_PSAD_* was amplified from pLM004-Venus using primers MOP830/MOP831 and inserted between the *Stu*I and *Nde*I sites of pMO449.

Plasmids like pMO449 but containing various IRES elements between the two ORFs were constructed as follows. For pMO455 (EMCV), a backbone was PCR-amplified from pMO449 using MOP658/MOP666 and assembled with an insert amplified from pIRES2-EGFP (Clontech) using MOP655/MOP665. For other plasmids, a fragment amplified from pMO449 using MOP658/MOP678 was assembled with fragments assembled from the indicated oligonucleotides: pMO467 (CrTMV cp), MOP686, MOP687, MOP688, and MOP689; pMO463 (PVY long), MOP679, MOP680, and MOP681; pMO464 (PVY short), MOP679 and MOP680; pMO466 (PFBV), MOP684 and MOP685; pMO473 (PLRV), MOP700 and MOP683; and pMO474 (PLRV^mut11^), MOP700, and MOP701.

To construct pMO507 and pMO508, pMO449 (except for *P_H/R_*) was PCR-amplified using MOP800/MOP801 and then assembled with a PCR fragment containing either *P_PSAD_* (amplified using MOP802/MOP803 from pLM004-Venus) or *P_TUB2_* (amplified using MOP804/MOP805 from genomic DNA). To construct pMO490 and pMO520, a PCR fragment containing either *sfGFP* [amplified using MOP657/MOP766 from pGEM-sfGFP (a gift from Tomohiro Kubo and George Witman)] or *Chlamydomonas*-codon-optimized *CrmCHERRY* [amplified using MOP832/MOP833 from pBR9-mCherry (Rasala *et al.* 2013)] was coassembled with a fragment containing the *3FLAG* coding sequence (amplified from pMO449 using MOP765/MOP663) into the *Hpa*I site of pMO448. To construct pMO488 and pMO519, a PCR fragment containing either *CrVENUS* (amplified using MOP657/MOP761 from pMO449) or *CrmCHERRY* (amplified using MOP833/MOP834 from pBR9-mCherry) was inserted into the *Hpa*I site of pMO448.

Plasmids containing gene fusions were constructed as follows. For pMO459, a *Lifeact-CrVENUS-3FLAG* sequence was amplified from pMO424 (Avasthi *et al.* 2014) using MOP674/MOP658 and inserted into the *Hpa*I site of pMO448. For pMO482 (PRO1/Cre10.g427250; MOP757/MOP758), pMO483 (*IDA5*/Cre13.g603700; MOP741/MOP742), pMO484 (*VFL2/*Cre11.g468450; MOP473/MOP475), and pMO515 (*PMH1/*Cre03.g164600; MOP815/MOP816), fragments generated by PCR from genomic DNA were inserted into the *Hpa*I site of pMO449; in each case, the indicated gene is fused in-frame to *CrVENUS-3FLAG*. For pMO480, a CVIM (“CAAX-box”)-encoding sequence assembled from oligonucleotides MOP749/MOP750 was inserted at the *Stu*I site of pMO449.

### Transformation

In most cases, plasmids were digested with *Eco*RV to excise the expression cassette before transformation. In a few cases in which the cassette itself contained an *Eco*RV site(s), *Sca*I was used to linearize the plasmid at a site outside the cassette. After heat-inactivation (65°, 10 min) of the enzyme used, the digestion reaction was used directly for transformation.

Three different electroporation methods were used for transformation during this study. Some early experiments used essentially the method described by Zhang *et al.* (2014): 240 µl of cell suspension (2 × 10^8^ cells/ml in TAP medium plus 40 mM sucrose) at 16° were mixed with 10 µl containing 2 µg of digested plasmid and electroporated in a 4-mm-gap electroporation cuvette using a Bio-Rad Gene Pulser with Pulse Controller, with pulse parameters of 800 V, 25 µF, and infinite Ω. However, we obtained considerably higher transformation frequencies when the cells were suspended instead in CHES buffer (10 mM *N*-cyclohexyl-2-aminoethanesulfonic acid, pH 9.25, 40 mM sucrose, and 10 mM sorbitol) and electroporation was carried out using the Gene Pulser with parameters of 600 V, 25 µF, and 1000 Ω. Still better results were obtained with the third method, in which cells were grown to ∼8 × 10^6^ cells/ml, resuspended at ∼8 × 10^8^ cells/ml in CHES buffer at room temperature, and electroporated in a volume of 125 µl in a 2-mm-gap electrocuvette using a NEPA21 square-pulse electroporator (Bulldog Bio; Yamano *et al.* 2013), using two poring pulses of 250 and 150 V for 8 msec each, and five transfer pulses of 50 msec each starting at 20 V with a “decay rate” of 40% (*i.e.*, successive pulses of 20, 12, 7.2, 4.3, and 2.6 V). In all three methods, electroporated cells were immediately transferred to a 15-ml centrifugation tube containing 8 ml TAP plus 40 mM sucrose. After overnight incubation at 24° under low light, cells were collected by centrifugation and spread on TAP agar (2% w/v) plates containing 10–40 µg/ml paromomycin.

### Fluorescence microscopy

Venus-expressing strains were grown in TAP liquid medium without antibiotics for ≥2 d with daily dilutions. A final culture at ∼1.5 × 10^6^ cells/ml was concentrated by centrifugation, mounted on a thin pad of TAP + 1% low-melting agarose (SeaPlaque; FMC Corporation), and sealed with a coverslip (for wide-field imaging) or placed in a glass-bottom culture dish (for confocal imaging). Wide-field imaging was performed using a Nikon Eclipse 600-FN microscope equipped with an Apochromat 100×/1.40 NA oil-immersion objective lens, an ORCA-2 cooled CCD camera (Hamamatsu Photonics), and Metamorph version 7.5 software (Molecular Devices). Confocal imaging was performed using a Leica DMI6000B microscope equipped with a Yokogawa CSU10 spinning-disk confocal-scanner unit, a Leica HCX PL Apo 63×/1.4–0.6 oil CORR lambda blue objective, an Andor iXon camera, and SlideBook 6.0 software. Images were postprocessed using ImageJ (National Institutes of Health) and Photoshop (Adobe) software.

### Fluorescence-scanner and plate-reader analyses

For fluorescence-scanner analysis, cells were spotted on a TAP agar plate containing 10 µg/ml paromomycin, grown for 3–5 d, and imaged using a Typhoon Trio fluorescence scanner (GE Healthcare) with excitation and emission wavelengths of 532 and 555 nm (gain setting PMT 800), respectively, to detect Venus, and 633 and 670 nm (PMT 500), respectively, to detect the chlorophyll autofluorescence. The images were postprocessed using ImageJ and Photoshop software.

Cells for plate-reader analysis were grown without shaking in wells of a transparent 96-well microplate containing 150 µl of TAP medium without paromomycin. For measurement, 75-µl aliquots of the cultures were combined with 75 µl of fresh TAP in wells of a black-sided plate (Nunc; 265301), and fluorescence (F) and absorbance at 750 nm (A) were measured immediately using a Tecan Infinite 200 Pro microplate reader. Fluorescence readings were acquired with excitation and emission wavelengths of 515 and 550 nm for Venus, 488 and 530 nm for GFP, and 572 and 608 nm for mCherry, with bandwidths of 9 (excitation) and 20 (emission) nm and a manual gain of 150. For each well, F and A were first corrected for the average backgrounds for TAP medium as measured in a separate plate: F/A = (F – mean F_TAP_)/(A – mean A_TAP_). In figures, F/A of the wells are expressed as values relative to the average of the pMO448 control samples.

### Analysis of protein expression by Western blotting

Cells growing in 5 ml TAP medium were collected by centrifugation at 2000 × *g* for 2 min, and the pellets were stored at −80°. For processing, samples were thawed in 100 µl of ice-cold PNE buffer (10 mM phosphate, pH 7.0, 150 mM NaCl, 2 mM EDTA) supplemented with a complete protease-inhibitor cocktail (Roche; 11697498001), and cells were disrupted by vortexing with acid-washed glass beads. Next, 100 µl of ice-cold PNE buffer + 2% NP-40 was added, followed by a 10-min incubation on ice, and the extracts were cleared by centrifugation at 12,000 × *g* for 10 min at 4°; 30 µg of each total cell extract were analyzed by SDS-PAGE (12%) and Western blotting, using a mouse anti-FLAG antibody (Sigma; F1804) and an HRP-conjugated rabbit anti-mouse-IgG antibody (ICN Pharmaceuticals; 55564).

### Data availability

Strains, plasmids, and plasmid sequences are available upon request and will be deposited at the Chlamydomonas Resource Center. File S1 contains sequences of all DNA oligonucleotides used in this study.

## Results

### Comparison of two-promoter and one-promoter expression systems for Chlamydomonas

**Figure 2:**
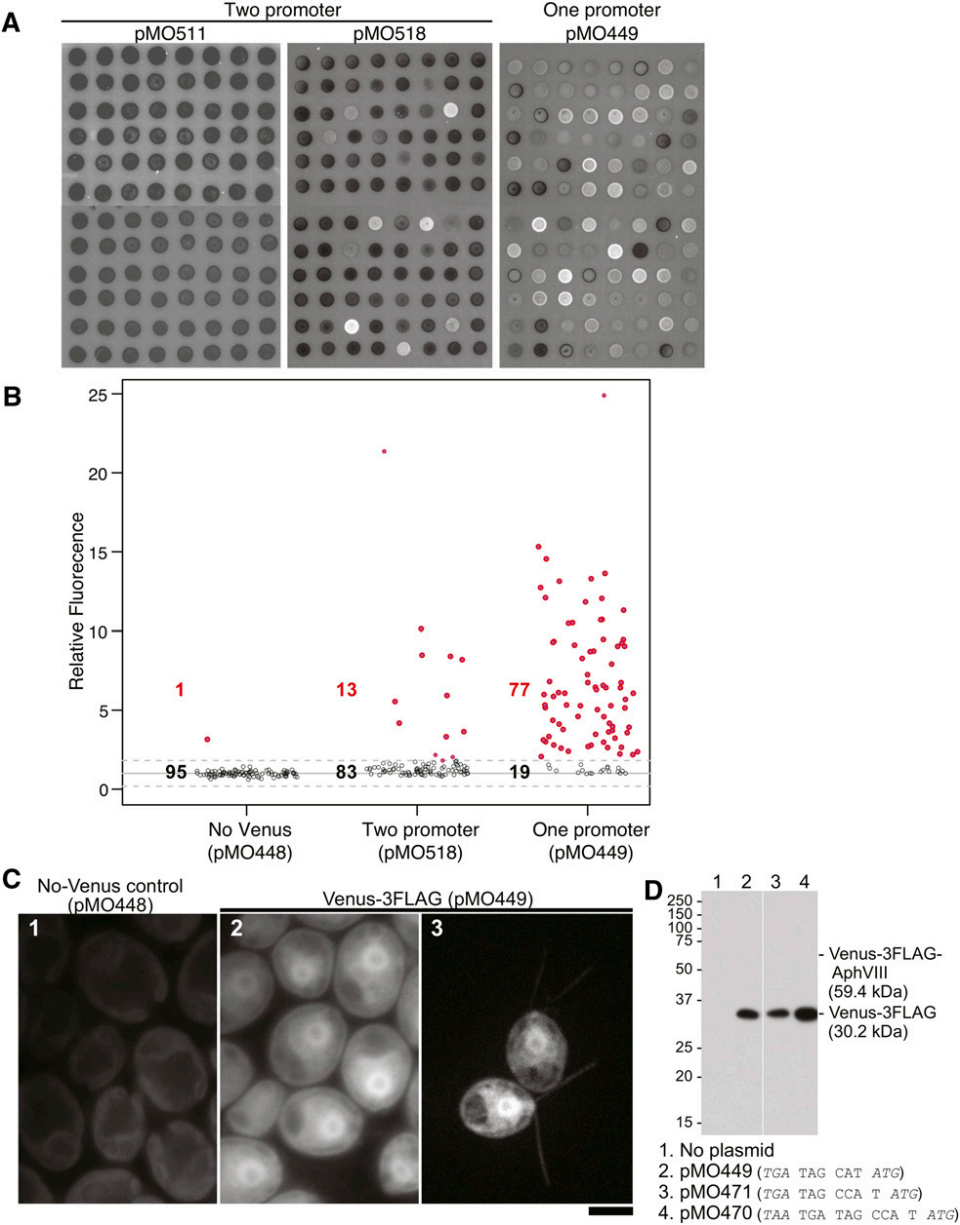
Frequencies and levels of Venus expression after transformation of *Chlamydomonas* with various constructs. (A and B) Strain CC-124 was transformed with the expression construct from plasmid pMO448 (control; no *VENUS* gene), pMO511 or pMO518 (two-promoter constructs; see Figure 1A), or pMO449 (one-promoter construct; see Figure 1B), and transformants were selected initially on 10 µg/ml paromomycin. In each case, 96 randomly chosen clones were analyzed further. (A) The clones from the pMO511, pMO518, and pMO449 transformations were spotted on plates containing 10 µg/ml paromomycin, grown for 3 d, and imaged using a fluorescence scanner (see *Materials and Methods*). (B) The clones from the pMO448, pMO518, and pMO449 transformations were grown in liquid medium without paromomycin in wells of a microplate for 5 d and analyzed using a fluorescence plate reader (ex515/em550 nm; see *Materials and Methods*). Each individual reading was normalized to the mean value for the control (pMO448) transformants, which was set at 1.0. Solid and dashed lines show the mean ± 3 SDs for the pMO448 transformants. Open circles and black numbers denote transformants whose fluorescence values were within the ± 3 SD window; red circles and numbers denote transformants whose values were above this threshold. (C) Cells of strain CMJ030 (1 and 2) or CC-124 (3) were transformed with the expression constructs from pMO448 (control) or pMO449 and selected initially on 20 µg/ml paromomycin. The transformants were grown further and observed by fluorescence microscopy using either the Eclipse (1 and 2) or spinning-disk (3) microscope as described in *Materials and Methods*. Image in 3 is a maximum projection of the *z*-stack. Weak signal in 1 represents autofluorescence from the chloroplasts; Venus signal in 2 and 3 is seen diffusely in the cytoplasms and (for unknown reasons) more strongly in the nuclei. (D) Cells of strain CMJ030 were transformed with the expression constructs from the indicated plasmids, selected initially on 20 µg/ml paromomycin, and then grown for 2 d at 24° in liquid medium with 1 µg/ml paromomycin. From each culture, 30 µg of total cell extract was subjected to SDS-PAGE and Western blotting using an anti-FLAG antibody. The predicted molecular weights of Venus-3FLAG and of a hypothetical Venus-3FLAG-AphVIII fusion protein are indicated.

Conventional two-promoter constructs, in which the GOI and the selectable marker are expressed from separate promoters, have typically had poor efficacy in achieving expression of the unselected GOI in *Chlamydomonas* (see *Introduction*). We obtained similar results when we used two such constructs (Figure 1A) to transform cells with the gene for the fluorescent Venus protein. Although many paromomycin-resistant transformants were obtained, only a small minority of them displayed Venus fluorescence (Figure 2, A and B, and Table 2, row 2).

We speculated that a one-promoter system might be more effective. In this case, a single promoter upstream of the GOI drives expression of both it and a downstream selectable marker from a bicistronic mRNA (Figure 1B). This arrangement should generally prevent the isolation of transformants in which the selectable marker is integrated and expressed without the GOI, or in which the whole construct is integrated in a chromosomal location allowing only poor expression or susceptible to subsequent transcriptional silencing. Thinking that an IRES would promote translation of the selectable marker, we first tried transforming with constructs containing *CrVENUS* connected to the *APHVIII* selectable marker by various virus-derived IRES-containing junction sequences (Table 2, rows 3–8), all of which have been used successfully for one-promoter expression of nonviral proteins in other systems (see File S2). The results with five of these sequences were disappointing: the numbers of paromomycin-resistant transformants were ∼100-fold less than in a control, and very few of the selected transformants expressed detectable levels of Venus as judged by fluorescence microscopy (Table 2, rows 1 and 3–7). These results suggested that these IRES elements do not function in *Chlamydomonas* and that the small numbers of transformants had acquired paromomycin resistance through integration of a partially digested DNA fragment containing *APHVIII* downstream of an endogenous promoter.

In contrast, the construct containing the PLRV IRES yielded several-fold more transformants, and about half of these were Venus positive (Table 2, row 8). This IRES contains four potential start codons (two near its 5′ end, one in the middle, and one at the 3′ end) that are in frame with that of the selectable marker, as well as a GGAGAGAGAGG motif that is thought to be important for its IRES function (Jaag *et al.* 2003). However, when we replaced this motif with the sequence CCTCTCTCTCC, which dramatically reduces IRES activity in potato protoplasts (Jaag *et al.* 2003), there was little or no effect (Table 2, row 9). This result suggested that it was not the IRES activity of PLRV that accounted for its partial function, but rather its potential start codons, and perhaps the fact that two of them lay in close proximity to the stop codon of *CrVENUS-3FLAG*.

Accordingly, we next tested three constructs that contained only short linkers between *CrVENUS-3FLAG* and the *APHVIII* selectable marker; these linkers also contained one or two additional stop codons in frame with that of *CrVENUS-3FLAG* and positioned the *APHVIII* start codon just 6–10 nucleotides (nt) downstream of the *CrVENUS-3FLAG* stop codon, and 3 or 4 nt downstream of the most proximal stop codon (Table 2, rows 10–12). Remarkably, not only did these constructs yield 5- to 10-fold more transformants than the IRES-containing constructs, but >80% of them expressed Venus at significantly more than background levels (Figure 2, A–C, and Table 2, rows 10–12). Given the additional stop codon(s) and the fact that *CrVENUS-3FLAG* and *APHVIII* are out of frame in two of the constructs, it seemed unlikely that the translating ribosomes simply read through into *APHVIII* to produce a functional fusion protein that confers paromomycin resistance. Indeed, when we examined the proteins directly by Western blotting with an anti-FLAG antibody, only a protein of the predicted size of Venus-3FLAG could be detected, even after extended overexposure (Figure 2D). Thus, it appears that translation of *APHVIII* is mediated by post-termination ribosome reinitiation (Skabkin *et al.* 2013), although further investigation would be needed to be certain of the molecular mechanism (see *Discussion*).

It should be noted that the two two-promoter constructs examined here showed striking differences not only in the overall frequency of Venus-positive transformants but also in the expression strength observed (Figure 2A). Although some individual transformants with pMO518 displayed expression as high as those seen with the one-promoter constructs, albeit at much lower frequency (Figure 2, A and B), no such highly expressing transformants were recovered with pMO511 (Figure 2A and our unpublished data). It is not clear which of the several differences between the two constructs (Figure 1A) account(s) for these differences.

In summary, given both the higher frequency of transformants with detectable GOI expression and (in many cases) the higher levels of that expression, it appears that the use of one-promoter constructs should greatly facilitate the identification of transformants suitable for a variety of downstream applications.

### Effect on expression strength of the antibiotic concentration used for selection of transformants

In one-promoter systems, a positive correlation would normally be expected between the expression levels of the two coexpressed proteins. With IRES-based expression vectors, this property has been utilized for screening and enrichment of cells with strong GOI expression, for example by fluorescence-activated cell sorting of transformants for high levels of expression of the marker gene downstream of the IRES (Liu *et al.* 2000). To ask if this would also be true for the *Chlamydomonas* expression constructs, we transformed wild-type cells with a one-promoter construct, a two-promoter construct, or a monocistronic *APHVIII*-only control, and incubated the transformants on plates containing various concentrations of paromomycin. As the antibiotic concentration was increased, a striking decrease of transformation efficiency was observed for the one-promoter construct, whereas the other constructs showed only modest decreases over the concentration range used (Table 3). These data suggest that the translation of *APHVIII* from the second cistron of a bicistronic mRNA is less efficient than from a monocistronic mRNA, so that relatively few transformants achieve sufficiently high expression for resistance to high paromomycin concentrations.

**Table 3:** Effect of paromomycin concentration on transformation efficiency

Plasmid*^a^*	Type	Relative Transformation Efficiency at Different Paromomycin Concentrations (µg/ml)			
		10	20	30	40
pMO449	One-promoter (see Figure 1B)	100	26	7.1	3.2
		100	34	11	3.6
		100	34	11	5.0
pMO518	Two-promoter (see Figure 1A)	100	97	87	83
		100	103	95	90
pMO448	Monocistronic (*APHVIII*-only) control (see *Materials and Methods*)	100	101	64	54
		100	93	76	71

a Data are shown from three (pMO449) or two (pMO518 and pMO448) separate transformation experiments using strain CC-124 and various electroporation methods (see *Materials and Methods*). In each experiment, the numbers of transformants per micrograms of DNA were normalized to the value obtained at 10 µg/ml paromomycin in that experiment.

**Figure 3:**
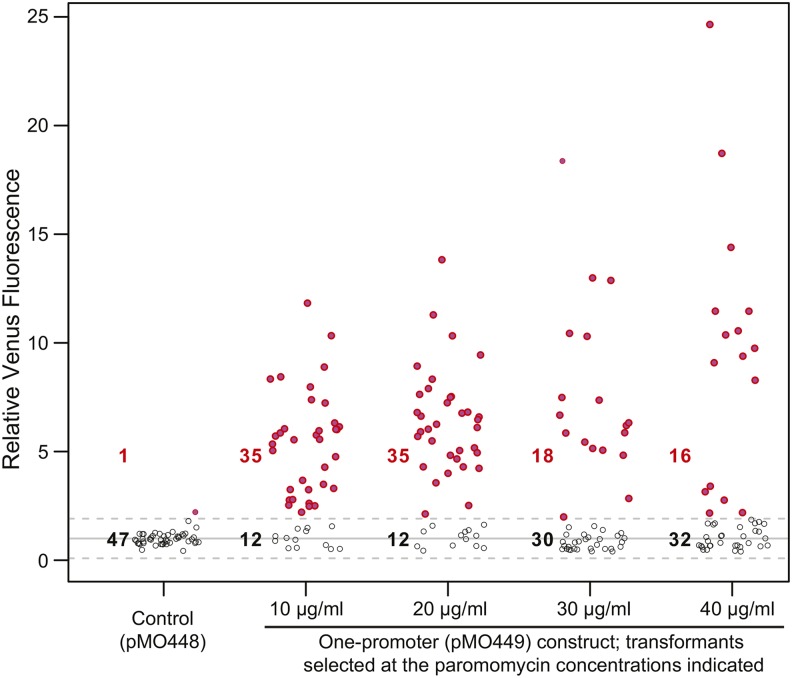
Effect of the paromomycin concentration used for selection on the fraction of GOI-expressing transformants and the level of GOI expression. Strain CC-124 was transformed with the one-promoter construct from pMO449, and transformants were selected on plates containing the indicated concentrations of paromomycin. Transformants with the non-GOI control plasmid pMO448 were selected on 10 µg/ml paromomycin. For each condition, 48 randomly chosen transformants were analyzed using a fluorescence plate reader as in Figure 2B.

Analysis of the transformants from the one-promoter construct for Venus fluorescence revealed two effects of increasing paromomycin concentration. First, at higher drug concentrations, the proportion of Venus-positive clones among the transformants decreased (Figure 3). It seems likely that this decrease occurred because relatively few of the transformants that actually expressed *APHVIII* via the bicistronic mRNA achieved sufficiently high levels of expression for resistance to high paromomycin concentrations, so that a larger fraction of the transformants recovered expressed *APHVIII* by some other mechanism such as integration of *APHVIII* by itself downstream of an endogenous promoter. Second, the Venus-positive clones that were recovered showed a moderate positive correlation between expression level and the antibiotic concentration that had been used to select them (Figure 3, Pearson’s *r* = 0.365), suggesting that the expression levels of the two proteins are indeed correlated (if somewhat weakly). This correlation probably reflects integration of the construct (containing both genes) at sites that allow different levels of transcription of the bicistronic mRNA. Taken together, the data suggest that when high levels of expression of a GOI are needed, it may be best to use a high antibiotic concentration for selection of the transformants; in this case, both a highly efficient transformation and a facile secondary screen for the transformants of interest would be desirable. When high levels of GOI expression are not required, it will probably be preferable to select transformants using a lower antibiotic concentration as determined empirically depending on the strain; we have successfully used as low as 5 µg/ml paromomycin with CC-124.

### Effects of altering the elements in the expression constructs

**Figure 4:**
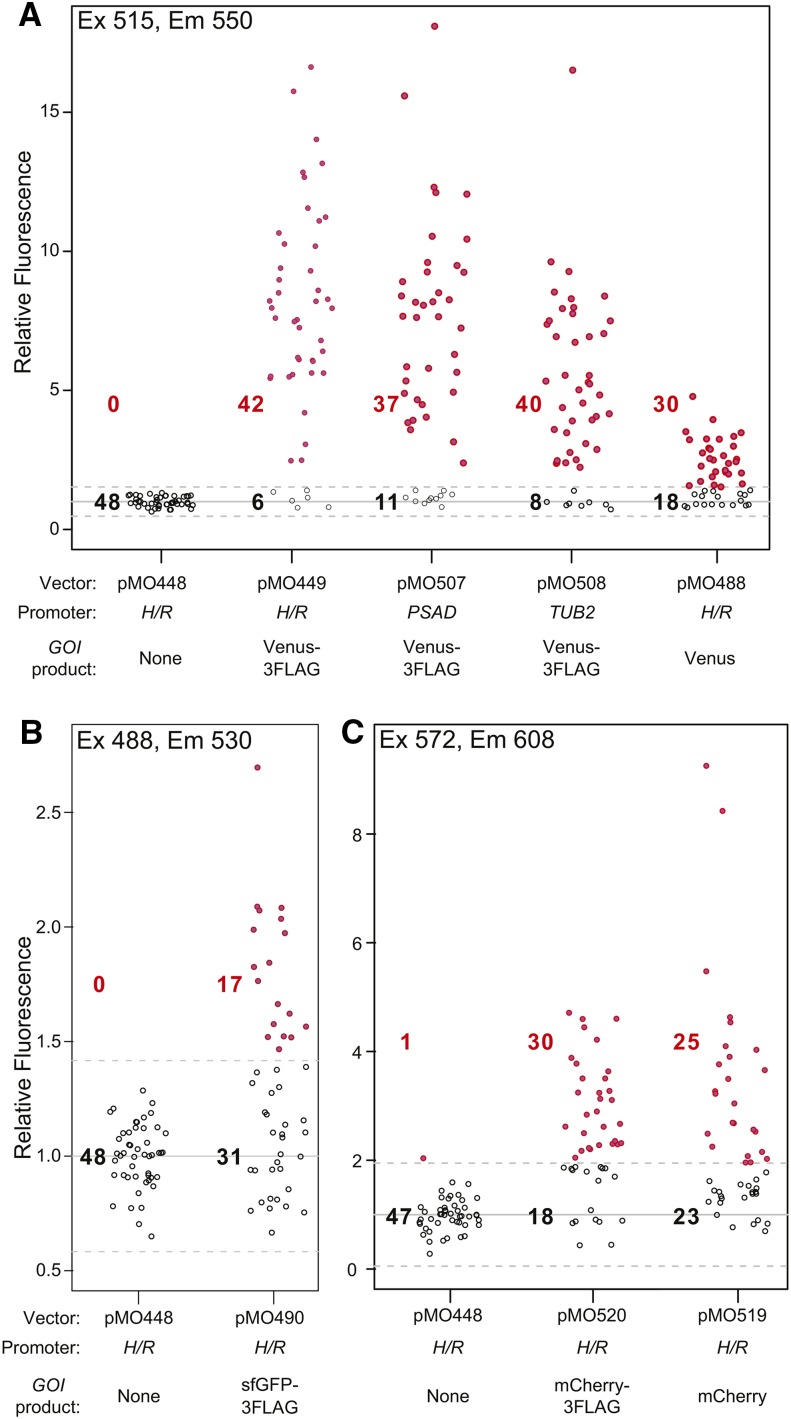
Successful use of one-promoter constructs with various promoters and GOIs. Strain CC-124 was transformed with the expression constructs from the indicated plasmids. In each case, 48 randomly chosen transformants were analyzed using a fluorescence plate reader as in Figure 2B, except that the excitation and emission settings appropriate for each fluorescent protein were used, as indicated. (A) Expression of Venus constructs. (B) Expression of sfGFP constructs. Note that the expression appears weak in the plot because of the high autofluorescence of *Chlamydomonas* cells in this spectral range. (C) Expression of mCherry constructs.

We next tested the effects of replacing various elements in the expression constructs. First, we replaced *P_H/R_* in pMO449 with either of two other widely used strong promoters, *P_PSAD_* and *P_TUB2_*, yielding pMO507 and pMO508. (These constructs retain the first three codons and intron of *RBCS2*, whose possible contribution to expression has not been tested.) When tested in parallel, the three promoters yielded similar fractions of Venus-positive transformants and similar levels of Venus expression under the conditions used (21°; 75 μmol photons m^−2^ sec^−1^ of constant light) (Figure 4A). Because these promoters respond differently to environmental perturbations (Davies *et al.* 1992; Fischer and Rochaix 2001; Schroda *et al.* 2002; Heitzer and Zschoernig 2007), the conditions to be used may determine the best promoter to use in expressing a transgene for a particular application.

Second, we tested the efficacy of the expression system with different GOIs. In addition to Venus-3FLAG, an untagged Venus (Figure 4A, pMO488), sfGFP-3FLAG (Figure 4B, pMO490), mCherry-3FLAG (Figure 4C, pMO520), and mCherry (Figure 4C, pMO519) were successfully expressed. Importantly, all of these genes had been codon-optimized for *Chlamydomonas*, and we failed to obtain GOI-expressing transformants using genes codon-optimized for other organisms (*EGFP* and *mCHERRY*, mammals; *yEGFP*, yeast) (data not shown). This failure probably reflects a need for efficient translation of the 5′ cistron in order to achieve efficient post-termination reinitiation for expression of *APHVIII*, so that the transformants actually recovered carry *APHVIII* alone (see also preceding section). Importantly, the recovery of transformants expressing Venus or mCherry without the 3FLAG tag indicates that nothing in the *3FLAG* coding sequence functions as a cryptic IRES or an essential element for post-termination reinitiation, as is seen in some viruses (Powell *et al.* 2008). Interestingly, the modest reduction in the average expression of Venus (pMO488) relative to that of Venus-3FLAG (pMO449) (Figure 4A) suggests that, in the pMO449 construct, the *3FLAG* sequence is actually somewhat inhibitory of *APHVIII* expression; thus, in the absence of this sequence, transformants can be recovered that have sufficiently high AphVIII expression for paromomycin resistance, even though expression of the bicistronic mRNA (and hence of the 5′ GOI) is at a lower level. Alternatively, it is possible that the stability and/or folding of Venus may be somewhat improved by the 3FLAG tag. Because *CrVENUS* and *CrmCHERRY* are of different origins, and have distinct nucleic-acid sequences (∼52% overall identity, ∼39% within the 120 nt upstream of the stop codons), the successful expression of both genes without a common *3FLAG* tag suggests that the RNA-sequence context immediately upstream of the intergenic region is not critical for the function of the constructs described here.

**Figure 5:**
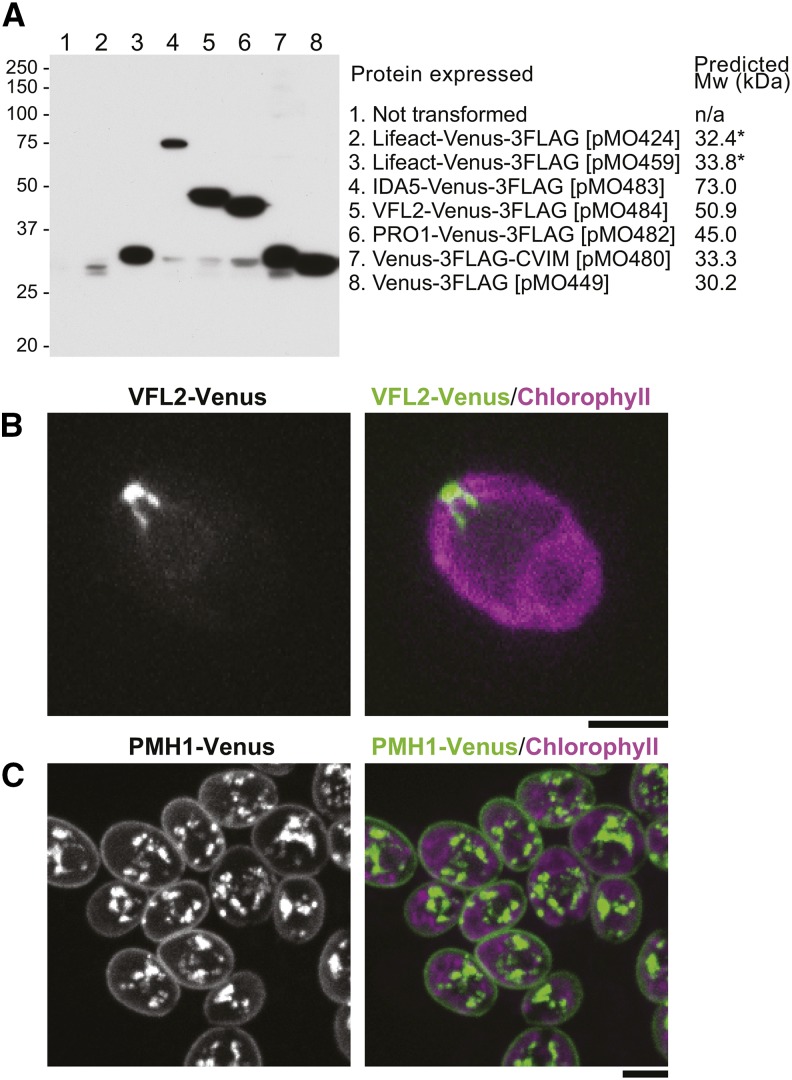
Expression of Venus-3FLAG-tagged peptides and proteins from one-promoter constructs. Strain CC-124 was transformed with the constructs from the indicated plasmids. (A) Analysis of protein expression by SDS-PAGE and Western blotting using an anti-FLAG antibody as described in Figure 2D. In all cases but pMO424, one transformant for each construct was chosen at random for analysis. For pMO424, 192 transformants were screened by fluorescence microscopy, and the one with the strongest signal was picked for further analysis. Predicted molecular weights of the fusion proteins are shown. *, the 1.4-kDa difference in predicted molecular weight of Lifeact-Venus-3FLAG expressed from the pMO424 and pMO459 constructs is due to the seven additional amino acids (MARRFEV) in the latter encoded by sequences added during the construction. (B, C) Localization of Venus-3FLAG-tagged VFL2 [(B); the same transformant as in (A)] and PMH1 [(C); transformation with the construct from pMO515]. Cells were observed using the spinning-disk microscope. Bars, 5 µm.

A particularly useful class of transgenes is those expressing a protein of interest tagged with a fluorescent protein, so we checked to be sure that the one-promoter constructs would give efficient recovery of transformants expressing such genes. Indeed, we were able easily to recover transformants showing good expression of either the tagged Lifeact peptide or any of several full-length proteins carrying a C-terminal Venus-3FLAG tag, as well as of Venus-3FLAG carrying a C-terminal CVIM (“CAAX-box”) peptide, as shown by Western blotting (Figure 5A), observations of *in vivo* fluorescence (Figure 5, B and C), or both. Note that although we screened many transformants with the two-promoter construct from pMO424 to find the one with the strongest expression of Lifeact-Venus, the expression level obtained (Figure 5A, lane 2) was considerably less than that in a randomly picked transformant obtained with the one-promoter construct from pMO459 (Figure 5A, lane 3). The intracellular localizations observed for VFL2 (Figure 5B), and PMH1 (Figure 5C), as well as for Lifeact, IDA5, and PRO1 (data not shown), were consistent with expectations (Taillon *et al.* 1992; Kato-Minoura *et al.* 1998; Kovar *et al.* 2001; Onishi *et al.* 2016), although Venus-3FLAG-CVIM failed to localize to the plasma membrane for unknown reasons. Interestingly, with both *IDA5-CrVENUS-3FLAG* and *VFL2-CrVENUS-3FLAG*, successful expression was achieved only when the full genomic coding sequences (*i.e.*, all exons and introns) were used, whereas the corresponding cDNA sequences failed even though they were inserted at the *Hpa*I site just downstream of the *RBCS2* intron (see Figure 1B).

Finally, we attempted to replace *APHVIII* in pMO449 with other antibiotic-resistance markers that have been used previously for selection of transformants in *Chlamydomonas*: *AadA* (spectinomycin resistance, codon-optimized; a gift from T. Yamasaki, Kochi University of Technology), *ble* (zeocin resistance; Lumbreras *et al.* 1998), and *aph7″* (hygromycin B resistance; Berthold *et al.* 2002). The *ble*-containing construct did not yield any transformants (data not shown). The *AadA*- and *aph7″*-containing constructs yielded small numbers of transformants when low antibiotic concentrations were used (30 µg/ml spectinomycin or 5 µg/ml hygromycin B), but none of the colonies was Venus positive (data not shown). It is not clear why these constructs failed, but it seems possible that these markers simply require higher expression than AphVIII to render the cells drug resistant.

## Discussion

We describe here a new method for expression of transgenes in *Chlamydomonas* in which a single promoter drives expression of a bicistronic mRNA containing two separate ORFs: an upstream GOI and a downstream paromomycin-resistance marker. With this system, we found that most paromomycin-resistant transformants also expressed the unselected GOI. The plasmids developed to date for applying this method contain one of three promoters, one of several cassettes for tagging proteins of interest, and convenient restriction sites for insertion of GOIs for tagging and/or overexpression (Table 4).

**Table 4:** Plasmids available for transgene expression using the one-promoter system

Plasmid*^a^*	Promoter*^b^*	Upstream Gene*^c^*	Selectable Marker*^d^*
pMO449*^e^*	*HSP70A/RBCS2*	*CrVENUS-3FLAG*	*APHVIII*
pMO470*^e^*	*HSP70A/RBCS2*	*CrVENUS-3FLAG*	*APHVIII*
pMO471*^e^*	*HSP70A/RBCS2*	*CrVENUS-3FLAG*	*APHVIII*
pMO488	*HSP70A/RBCS2*	*CrVENUS*	*APHVIII*
pMO490	*HSP70A/RBCS2*	*sfGFP-3FLAG*	*APHVIII*
pMO519	*HSP70A/RBCS2*	*CrmCHERRY*	*APHVIII*
pMO520	*HSP70A/RBCS2*	*CrmCHERRY-3FLAG*	*APHVIII*
pMO507	*PSAD*	*CrVENUS-3FLAG*	*APHVIII*
pMO508	*TUB2*	*CrVENUS-3FLAG*	*APHVIII*
pMO561*^f^*	*TUB2*	*CrVENUS-3FLAG*	*APHVIII*

a Available plasmids with one-promoter expression constructs of the general structure shown in Figure 1B.

b Except as indicated in note *^f^*, all promoters are followed by the first three codons and intron of *RBCS2*.

c All genes indicated have been codon optimized for *Chlamydomonas*. These genes can be replaced *in toto* by *Hpa*I–*Stu*I fragments containing other GOIs whose expression/overexpression is desired. Fluorescence tagging of a gene product of interest can be achieved by inserting the coding sequence, in frame, at the *Hpa*I site (C-terminal tagging) or the *Stu*I site (N-terminal tagging).

d Other selectable markers could be inserted as *Nde*I–*Bam*HI fragments. To date, however, we have not had success with selectable markers other than *APHVIII* (see text).

e These plasmids differ only in the short linker between *CrVENUS-3FLAG* and *APHVIII* (see Figure 2D and Table 2).

f Same as pMO508 except that the *RBCS2* ATG start codon has been changed to TTG. This allowed expression of *CrVENUS-3FLAG*, presumably from its own start codon (*i.e.*, without the additional amino acids from *RBCS2*).

When Venus was used as a GOI with this system, a positive correlation was found between paromomycin resistance and Venus fluorescence, indicating that expression from the two ORFs is coupled. It initially seemed possible that a mechanism such as stop-codon read through (Jackson *et al.* 2012) might result in expression of the GOI and *APHVIII* products as a single fusion protein that provides paromomycin resistance. However, this appears not to be the case, because the method works equally well when two or three different stop codons are inserted between the ORFs, and/or the ORFs are placed out-of-frame, and Western blotting detected no such fusion products. Thus, it seems most likely that *APHVIII* is translated by post-termination reinitiation (Kozak 2007; Skabkin *et al.* 2013), in which the ribosome remains associated with the mRNA after termination, continues scanning, and reinitiates translation at a downstream (or, occasionally, upstream: Skabkin *et al.* 2013) AUG. Such events have been well documented for transcripts that have naturally occurring upstream ORFs (uORFs) in many organisms (Wang and Wessler 1998; Kozak 2007; Hinnebusch and Lorsch 2012), and many annotated transcripts in the *Chlamydomonas* genome also have uORFs, although it is not yet known how many of these are actually translated (Cross 2016). Most uORFs in these cases are very short (*e.g.*, 3–4 codons for yeast *GCN4* and 38 codons for maize *R*), and a negative correlation between uORF length and reinitiation efficiency at the downstream AUG has been reported (Kozak 2007). Thus, it is somewhat surprising that the long “uORFs” in the one-promoter constructs used in this study (*e.g.*, 654 codons for *IDA5-CrVENUS-3FLAG*) allowed effective translation of the downstream *APHVIII*.

Indeed, it appears that the translation of *APHVIII* is less efficient than that of the upstream GOI, given that most transformants did not grow at a paromomycin concentration of 40 µg/ml (Figure 3 and Table 3), whereas most transformants obtained with constructs expressing *APHVIII* from a monocistronic mRNA were able grow at 40 µg/ml (Table 3), or even 80 µg/ml (our unpublished data), paromomycin. Enhancing translation of the downstream selectable marker might potentially enable the use of markers other than *APHVIII*. However, our attempts to date with virus-derived IRES-containing sequences failed to achieve this goal. We also considered the possibility that the 5′ portion of *APHVIII* contains a cryptic IRES-like translation enhancer activity. However, joining the first 120 nt of *APHVIII* to other markers (*aph7″*, *ble*, and *AadA*) still did not yield any Venus-positive clones with these markers (data not shown). It is possible that using other viral IRESs or endogenous *Chlamydomonas* sequences from bicistronic (Cenkci *et al.* 2003) or uORF-containing (Cross 2016) transcripts would be more successful.

The one-promoter system has significant advantages over traditional two-promoter strategies, of which the chief is clearly the high percentage of transformants that express the GOI. This feature should be particularly valuable in studies in which a given marker needs to be expressed in multiple strains. For example, in our initial attempts to use Lifeact-Venus to determine actin localization in vegetative cells (Avasthi *et al.* 2014), we needed to screen >100 transformants to isolate a clone with a barely sufficient level of expression (see also Figure 5A). In contrast, a one-promoter construct allowed us to quickly and easily observe Lifeact-Venus localization in multiple strains, and to screen mutants for altered F-actin structures (Onishi *et al.* 2016; our unpublished data). It should be possible to perform similar expression-based screens with other biomarkers or other genes of scientific or industrial importance. The efficient recovery of GOI-expressing transformants should also greatly facilitate mutagenesis-based functional analysis of GOIs.

Second, although some two-promoter constructs yield individual transformants with high levels of GOI expression, others do not (Figure 2, A and B, and Figure 5A), so that when high levels of expression of a GOI are desired, a one-promoter construct seems likely to provide the method of choice.

Finally, because translation of the GOI should be a prerequisite for that of *APHVIII*, propagation of transformants in the presence of paromomycin should circumvent the silencing of GOIs after repeated passages that has sometimes been observed (Cerutti *et al.* 1997). [However, we have also observed that, at least in most cases, paromomycin can be omitted from the medium for ≥2 wk without affecting the levels of GOI expression observed, an approach that may often be desirable given the known effects of paromomycin in decreasing the fidelity of translation (Davies *et al.* 1965; Mironova *et al.* 1982; Hirokawa *et al.* 2007).] Moreover, if a particular GOI fails to express efficiently for any reason, the number of transformants obtained would simply drop, saving the investigator from screening hundreds of colonies for a nonexistent GOI-expressing clone. These considerations also highlight the importance of optimizing the codons of heterologous genes to be expressed in *Chlamydomonas*. This seems likely to be even more important with the one-promoter system than it is with two-promoter constructs (Cerutti *et al.* 1997; Fuhrmann *et al.* 1999), and indeed we have failed to recover transformants with several heterologous genes that had not been codon optimized (our unpublished data).

Interestingly, we failed to express *IDA5* and *VFL2* when cDNAs were inserted at the *Hpa*I site of pMO449 (Figure 1B), although insertion of the full genomic copies of the genes (with introns) yielded transformants with good levels of expression (Figure 5). As all of these constructs contained the first intron of *RBCS2*, which is known to enhance transcription (Lumbreras *et al.* 1998), it is not clear why the additional introns of *IDA5* and *VFL2* improved expression. We have also failed to express some other *Chlamydomonas* and heterologous genes using the one-promoter system, presumably either because their ectopic overexpression is toxic to cells, or because their translation efficiency is insufficient to support adequate expression of the downstream *APHVIII* through translation reinitiation.

The expression system described here is distinct from a previously described one-promoter system based on the viral 2A peptide (Rasala *et al.* 2012). In the 2A system, the transformants express a single mRNA encoding the *ble* selection marker, the viral 2A peptide, and a GOI. When a translating ribosome encounters the 2A sequence, no peptide bond is formed between the last two amino acids of the 2A peptide, but the ribosome continues to translate the downstream coding sequence, thereby achieving coexpression of two (or more) proteins from a single mRNA. However, the failure of peptide-bond formation at the 2A peptide is sometimes incomplete, so that a small proportion of the marker and GOI-encoded protein may be expressed as a fusion protein (Geu-Flores *et al.* 2008; Rasala *et al.* 2012; Kong *et al.* 2015), potentially impairing the function of the GOI product or otherwise complicating interpretation of the results obtained. Moreover, the coexpression efficiency of the GOI with the *ble* marker seems to be low, so that a PCR-based screen for transformants that received a complete cassette, and subsequent assays for strong expression, have been performed in studies using the 2A technique (Rasala *et al.* 2012, 2014; Kong *et al.* 2015). However, the 2A system also has an advantage over the system described here, in that it can express multiple proteins from a single transformed construct (Rasala *et al.* 2014), whereas this is not feasible with the system described here due to the inefficiency of translation reinitiation. It seems likely that each system will be useful in appropriate applications in the future.

In summary, we have described here a one-promoter, bicistronic-mRNA system for expression of transgenes in *Chlamydomonas* that should greatly facilitate a wide variety of experiments. Although only a limited set of plasmids has been constructed to date (Table 4), others (*e.g.*, constructs containing other promoters or markers for gene tagging) could easily be constructed using the backbone provided by pMO449 (Figure 1B). A limitation of the system to date is the availability of only a single selectable marker; it should be possible to overcome that limitation in future studies.

## Supplementary Material

Supplemental Material
