# Supplementary material for: Robust Transgene Expression from Bicistronic mRNA in the Green Alga Chlamydomonas reinhardtii
Source: G3 (Bethesda). 2016 Oct 20;6(12):4115–25. doi: 10.1534/g3.116.033035 (PMC5144980; doi:10.1534/g3.116.033035)
Supplement: Supplemental Material [file supp_6_12_4115__index.html]

Robust Transgene Expression from Bicistronic mRNA in the Green Alga Chlamydomonas reinhardtii — Supplemental Material 

# Robust Transgene Expression from Bicistronic mRNA in the Green Alga *Chlamydomonas reinhardtii*

## Supplemental Material for Onishi, *et al*, 2016

**Files in this Data Supplement:**

- File S1 - Primers used in this study. (.xlsx, 12 KB)
- File S2 - IRES. (.xlsx, 36 KB)
